# Septal agenesis and lissencephaly with colpocephaly presenting as the ‘Crown Sign’

**DOI:** 10.4103/1817-1745.76106

**Published:** 2010

**Authors:** Namit Singhal, Sunil Agarwal

**Affiliations:** Department of Neurosurgery, Heritage Hospital, Agra, India; 1 Pusphanjali Hospital, Agra, India

**Keywords:** lissencephaly, colpocephaly, septal agenesis, crown sign

## Abstract

Developmental malformations of the cortex in neuronal migration disorders result in constellation of findings on radiological scanning. Isolated defects are common but sometimes they occur in varying degrees of combination giving a unique appearance on the imaging studies. We describe a case of neuronal migration disorder in which the computed tomography scan showed the presence of lissencephaly, colpocephaly. and Septal agenesis. These findings make the ventricular system appear in shape of a crown, which we refer to as “CROWN SIGN”, first described in neurosurgical literature..

Neuronal migration disorders refer to a wide spectrum of developmental malformations of the cortex caused by disruption to its normal process of formation, which includes proliferation, migration and organization. We illustrate case of a 2 year old male child who presented to us with complaints of delayed milestones of development since birth with multiple episodes of generalized tonic clonic seizures. Physical examination revealed a depressed anterior fontanelle. Computed tomography scan showed the presence of lissencephaly, colpocephaly and septal agenesis. This constellation of findings makes the ventricular system appear in shape of a crown of a king on the un-enhanced CT scan. Hence, we refer to this as “CROWN SIGN”, not previously described in neurosurgical literature. The child was started on anti-epileptics and cerebro-active agents. He showed gradual improvement on follow up with neck holding coming up as the first positive sign. Isolated defects are common but sometimes they occur in varying degrees of combination, giving a unique appearance on the imaging studies, as has been exemplified in our case.

A 2-year-old male child presented to us with complaints of delayed motor, language, and psychosocial milestones of development since birth. At the time of examination, there was no neck holding. For the last six months, the child also had multiple episodes of generalized tonic-clonic seizures. The perinatal period was insignificant. There was no positive family history. Physical examination revealed a depressed anterior fontanelle. Computed tomography (CT) scan was done, which showed the presence of lissencephaly (pachygyria-agyria complex) [Figure 1] with predominant enlargement of occipital horns (colpocephaly) [Figure 2]. Septal agenesis was also present, although the interhemispheric fissure was well developed. Dandy-Walker variant was present as an additional finding [Figure 3]. This triad of lissencephaly, colpocephaly, and absence of septum pellucidum gives a unique appearance to the ventricular system in form of a crown of a king on the unenhanced CT scan. Therefore, we refer to this as ‘CROWN SIGN,’ first described in neurosurgical literature [Figure 4]. This terminology has been used to provide a quick insight about the pathology that is being dealt with. The presence of ‘CROWN SIGN’ indicates that we are dealing with syndromic triad of lissencephaly, colpocephaly, and septal agenesis. The child was started on antiepileptics and cerebroactive agents. He showed gradual improvement on follow-up, with neck holding coming up as the first positive sign.

**Figure 1:**
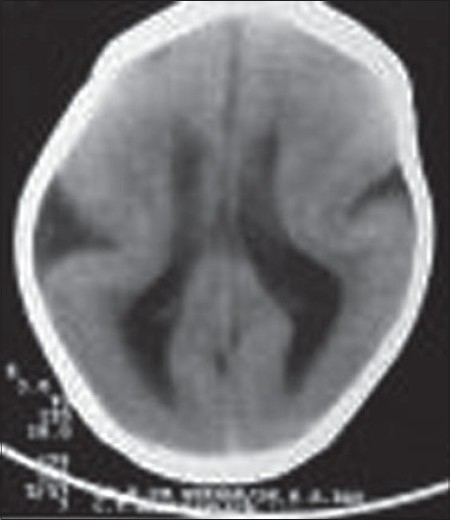
Noncontrast axial CT scan showing the presence of lissencephaly with pachygyria-agyria complex

**Figure 2:**
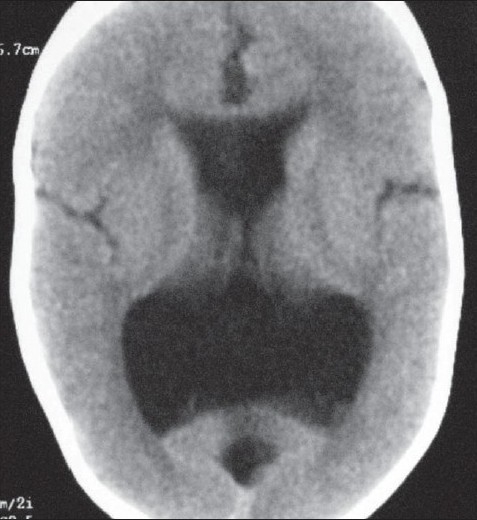
Noncontrast axial CT scan showing the presence of colpocephaly with septal agenesis

**Figure 3:**
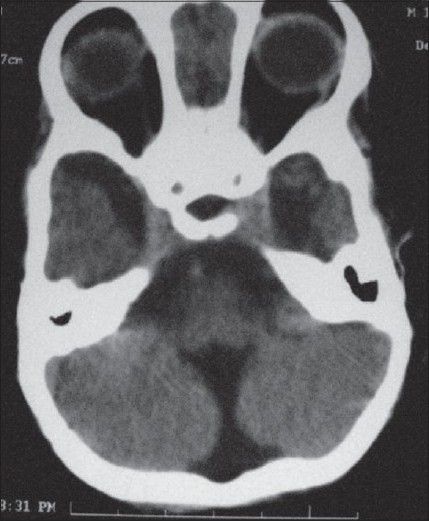
Dandy-Walker variant presenting as the ‘key sign’

**Figure 4:**
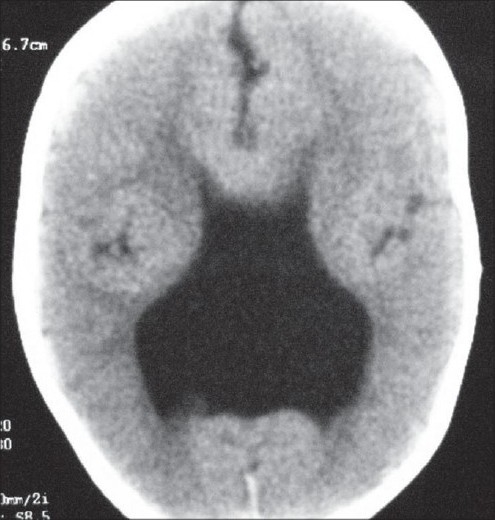
Ventricular system presenting as the ‘Crown Sign’

## Discussion

Neuronal migration disorders (also, and better, called cortical developmental anomaly) are caused by abnormal proliferation, migration, and organization (lamination, gyration, and sulcation). Proliferation of young neurons occurs in the germinal matrix, located in the subependymal layer of the walls of the lateral ventricles, during the seventh week of gestation. Starting from the eighth week of gestation, most of these neurons migrate from the germinal zone to their final destination in the cortex along specialized radial glial fibers, 10% migrate orthogonally to the radial glial cells. Migration follows an ‘inside-out’ sequence so that neurons of the deepest cortical layer migrate early, followed by those destined for layers 5, 4, 3, and 2, with the exception of neurons destined for layer 1 that are the first to reach the cortex. Once in the cortex, neurons organize into the normal six layers and develop synaptic contacts with the other neurons. Any event that interferes with the various steps of cortical formation can cause a developmental anomaly. This includes infections (cytomegalovirus, toxoplasmosis), ischemic insults, both exogenous and endogenous toxins from metabolic disorders and radiation exposure.[1]

Cortical malformations manifest clinically by producing seizures, mental retardation, and focal neurological deficits. Most frequently, patients experience medically refractory epilepsy, whose degree of severity and time of onset is variable. Other features include feeding and swallowing problems, muscle tone anomalies (early hypotonia and subsequently limb hypertonia), and severe psychomotor retardation.[2,3] CT changes of lissencephaly include lack of cortical sulci and gyri; calcification in the region of paraphysis; wide, shallow sylvian fissures; colpocephaly; poor development of white matter; and persistent cavum septum pellucidum and cavum vergae.[4] Isolated defects are common but sometimes they occur in varying degrees of combination,[5] giving a unique appearance on the imaging studies, as has been exemplified in our case.
